# Selection, Characterization, and Application of ssDNA Aptamer against Furaneol

**DOI:** 10.3390/molecules23123159

**Published:** 2018-11-30

**Authors:** Natalia Komarova, Mariia Andrianova, Sergey Glukhov, Alexander Kuznetsov

**Affiliations:** Scientific-Manufacturing Complex Technological Centre, 1–7 Shokin Square, Zelenograd, 124498 Moscow, Russia; smariika1987@gmail.com (M.A.); serglukhovmb@gmail.com (S.G.); kae@tcen.ru (A.K.)

**Keywords:** aptamer, furaneol, Capture-SELEX, ISFET, aptasensor

## Abstract

Furaneol is an aroma compound which occurs naturally in foods and is used as an artificial flavor. Detection of furaneol is required in food science and food processing industry. Capture- Systematic Evolution of Ligands by EXponential enrichment (SELEX) protocol was applied for the isolation of an aptamer binding to furaneol, a small volatile organic substance contributing to the flavor of various products. Thirteen cycles of selection were performed. The resulting DNA pool was cloned, using blunt-end cloning, and ninety-six plasmids were sequenced and analyzed. Eight oligonucleotides were selected as aptamer candidates and screened for the ability to bind to furaneol, using three different methods—magnetic-beads associated elution assay, SYBR Green I assay, and exonuclease protection assay. One of the candidates was further characterized as an aptamer. The apparent equilibrium constant was determined to be (1.1 ± 0.4) µM, by the fluorescent method. The reported aptamer was applied for development of the ion-sensitive field-effect transistor (ISFET)-based biosensor, for the analysis of furaneol, in the concentration range of 0.1–10 µM.

## 1. Introduction

4-Hydroxy-2,5-dimethyl-3(2H)-furanone (Furaneol, HDMF) is an important flavor agent contributing to the aroma and taste of many natural products and thermally-processed foods. Furaneol has a sweet, caramel-like, fruity smell that is believed to be associated with a planar enol-oxo group of cyclic dicarbonyl compounds [1]. Furaneol might significantly contribute to the overall flavor, due to its low threshold values for human [1]. Furaneol has been detected in wines [2,3], coffee [4,5,6], fruits like strawberry, raspberry or pineapple [7,8,9], and tomatoes [7,10]. Furaneol is also formed during the heat processing of sugar-containing products [11]. Detection of furaneol is required in food analysis. It can also be used in the food industry, for example, for wine origin recognition [12] and control of brandy age [13], or process control during food and beverage production [14]. Furaneol is mostly detected using well-equipped techniques, like gas chromatography [10,15,16,17], assisted with preliminary extraction and concentration procedures [2,13,18].

Aptamers are single-stranded DNA or RNA molecules capable of binding specifically with a target molecule. Aptamers can serve as an analytical tool, replacing costly antibodies, and can be used as therapeutic or drug delivery agents [19]. Aptamers to various targets, including proteins, viruses, small organic molecules, and whole cells, have been isolated [20].

Aptamers to a desired target can be selected from a large, random, single-stranded DNA (ssDNA) or RNA library, using the SELEX method (Systematic Evolution of Ligands by EXponential enrichment) [21,22]. The DNA aptamer SELEX is an iterative process consisting of four steps: incubation, partition, amplification, and ssDNA regeneration. During incubation, the random ssDNA library is incubated with the target, and the oligonucleotides capable of binding to the target form a complex with it. During partition, the oligonucleotides bound to the complex are separated from the free DNA molecules, and the eluted DNA pool is then PCR-amplified, in the amplification step. In the last step, the ssDNA is regenerated from the double-stranded (ds) PCR product, producing an ssDNA pool that is enriched with sequences having higher affinities to the target, than the original ssDNA library. This pool is used in the next round of SELEX and the selection is continued until the ssDNA pool becomes sufficiently enriched with target-affine sequences. SELEX involves a lot of modifications aiming to improve the partition efficiency and allowing the minimization of the number of selection cycles. Most SELEX protocols assume immobilization of the target molecule onto a solid support; these methods are well-suited for selection of aptamers against large targets like cells or proteins [20]. Small-molecule targets are usually difficult to immobilize, and their immobilization can result in the loss of binding sites. Capture-SELEX protocol designed, especially, for small molecule SELEX, is notable for the reversible fixation of the ssDNA library onto the solid support, instead of the target immobilization [23]. The DNA library for the Capture-SELEX is extended, with a special capture-sequence inserted into the random region. This capture sequence serves for the hybridization of the ssDNA, on the complementary DNA probe, which, in turn, is covalently-attached to the surface of magnetic beads, via a biotin-streptavidin interaction. Thus, the library is fixed on the surface of magnetic beads and then incubated with a free target solution. During incubation, ssDNA molecules dissociate reversibly from the capture probe, and some of them can form a complex with the target molecules. Oligonucleotides that have passed to the solution are easily separated from the rest of the library, using a magnetic rack, and this eluted DNA pool is enriched with sequences that have an affinity to the target. This pool is PCR-amplified with fluorescently-labeled forward primer, and the ssDNA is regenerated for the next selection round. The fluorescent label enables a simple quantification of the ssDNA, starting from the second round of selection, and this helps to monitor the progress of the pool enrichment. This method of aptamer selection enables the isolation of the so-called structure-switching aptamers, assuming that in the presence of the target, the aptamer molecule undergoes a structural switch with the target, between the capture-probe duplex and the complex. The Capture-SELEX protocol has been successfully applied to obtain aptamers to small molecules, like different antibiotics [23,24,25,26], natural sweetener rebaudioside A [27], and flavoring agent vanillin [28].

The ion-sensitive field-effect transistor (ISFET) is a type of metal-oxide-semiconductor field-effect transistor (MOSFET) in which the metal gate is replaced by a liquid [29]. Adsorption occurring on the liquid-sensitive surface interface modulates the current in the transistor channel, and the current change may serve as an analytical signal. ISFET is a highly-sensitive transducer that can detect DNA duplex formation [30]. Several biosensors that are based on a combination of the ISFET and aptamers, have been described previously [28,31,32].

In this research, we report the isolation of the ssDNA aptamer, binding to furaneol, and its use for aptasensor development. The Capture-SELEX protocol was applied for the aptamer selection. Eight aptamer candidates were screened for their ability to bind to furaneol, using three different methods. The best candidate was characterized with an equilibrium dissociation constant and selectivity. This aptamer was immobilized on the sensitive surface of the ISFET and hybridized with a short complementary DNA probe. Upon addition of the target to the ISFET gate, the change in aptamer structure resulted in the probe dissociation that was detected by the transistor. The sensor could detect 0.1–100 µM of furaneol.

## 2. Results

### 2.1. Aptamer Selection

The selection of aptamer, against the furaneol, was carried out using the Capture-SELEX protocol designed by Stoltenburg et al. [23]. Few modifications were made, compared to the original protocol. A Cy3 fluorescent label was used for labeling the sense ssDNA strand, and volumes of the target solutions, during the incubation step, were decreased.

The original ssDNA library, named B1_bank, was used for the aptamer selection. The library was flanked by two primer binding sites of 18 and 19 nt, and a random fragment of 50 nt was separated to 10 and 40 nt, by a capture sequence of 12 nt. The capture sequence allowed the fixation of the library on the capture oligonucleotide. It was designed to display a high binding efficiency, during library hybridization, within the minimum length.

The capture probe B1_cap, complementary to a fragment of the B1_bank ssDNA library, was immobilized onto the magnetic support, via a biotin-streptavidin interaction, and then the B1_bank was hybridized on the capture probe. The beads, with the ssDNA library fixed on them, were incubated with 1 mM furaneol solution. Before each incubation with the target, negative selection against the selection buffer (SB), without the target, was performed, to estimate the background DNA elution and the affinity of the eluted pool, to furaneol. Target concentration, number of washes, and the elution time were kept constant, throughout the selection.

After incubation with the target, the solution with the eluted ssDNA was amplified by PCR with Cy3-labeled forward (B1_for_Cy3) and elongated reverse (B1_rev_A) primers, and the DNA strands were separated on the denaturing PAGE. The Cy3-labelled strand was then removed from the gel, extracted, and purified. The entire ssDNA pool was then evolved to the next SELEX round; the amount of ssDNA before each round is listed in Table 1.

**Figure 1:**
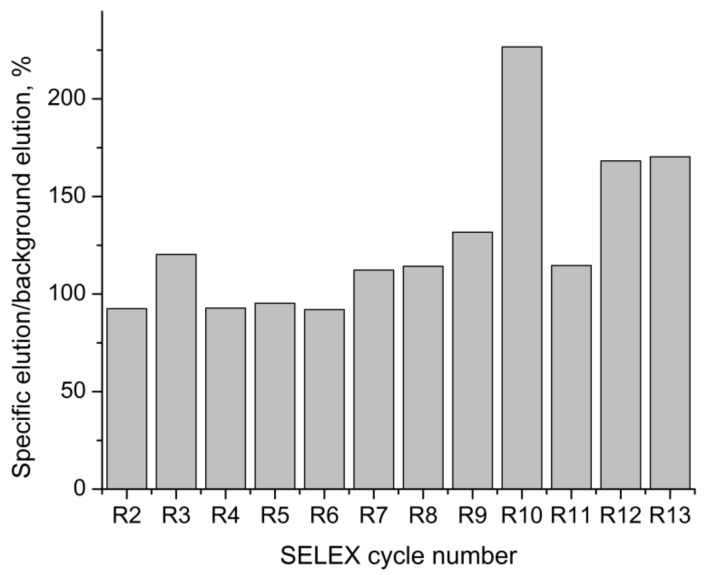
Ratio of the amount of ssDNA eluted by 1 mM furaneol (specific elution) to the amount of ssDNA eluted by the buffer (background elution), during the aptamer selection.

The amount of the eluted target ssDNA was quantified using Cy3 fluorescence. The amount of ssDNA eluted both by the buffer (background elution) and by the target (specific elution) was monitored during the selection (Table 1). Their ratio can be used for the monitoring of the aptamer selection progress [23]. At the starting rounds, when there were few oligonucleotides with specific affinity to the target in the eluted ssDNA pools, nearly the same amounts of DNA were eluted, both by the buffer and by the target solution. During the selection process, the DNA pool is enriched with aptamers, and in the presence of the target, more DNA is eluted from the capture probe, in comparison to background. When the ratio of the amount of specifically-eluted DNA to background elution stops increasing, the enrichment process is believed to have been finished [23]. The enrichment of the eluted DNA pool is presented on Figure 1. The maximum ratio of the specific elution by furaneol to background elution was observed during the tenth round. During the following cycles of selection, the amount of eluted DNA decreased, due to the lower yield of ssDNA regeneration (see Table 1), and the ratio of the specific to background elution also started decreasing. The selection was stopped at the thirteenth cycle.

**Figure 2:**
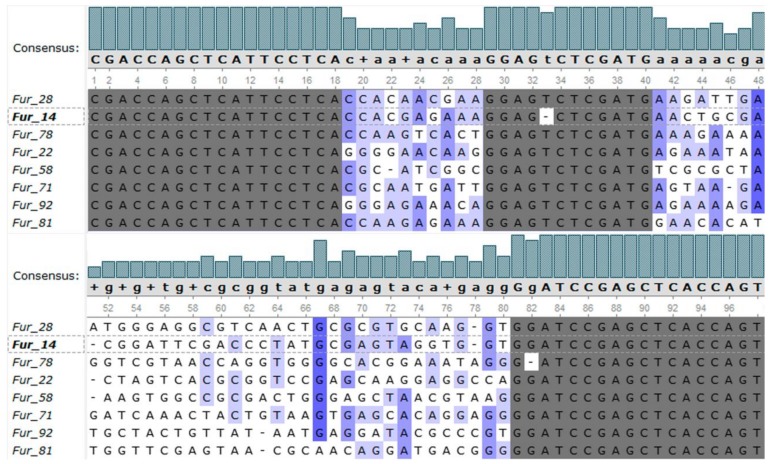
Alignment of the candidate aptamer sequences. Constant regions of the B1_bank library are highlighted in grey. Alignment was performed using Clustal Omega algorithm in the BioEdit software and visualized in the Unipro UGENE software.

The resulting ssDNA pool was cloned using the blunt-end ligation, and ninety-six plasmids were extracted and sequenced. Among them seventy-four plasmids had inserts with the structure of the initial B1_bank library. The inserts were aligned and analyzed for similarity. Each sequence of the pool was unique. The alignment of all sequences is provided in the Supplementary document, see Figure S1. A phylogenetic tree (see Figure S2) was built. The phylogenetic tree had three orphans and eight clusters counting 4, 8, 9, 9, 10, 13, and 18 sequences each. One sequence from each cluster was selected as the aptamer candidate; in total eight sequences were selected. The alignment of the candidate aptamer sequences is presented in Figure 2.

### 2.2. Screening of the Aptamer Binding Ability

**Figure 3:**
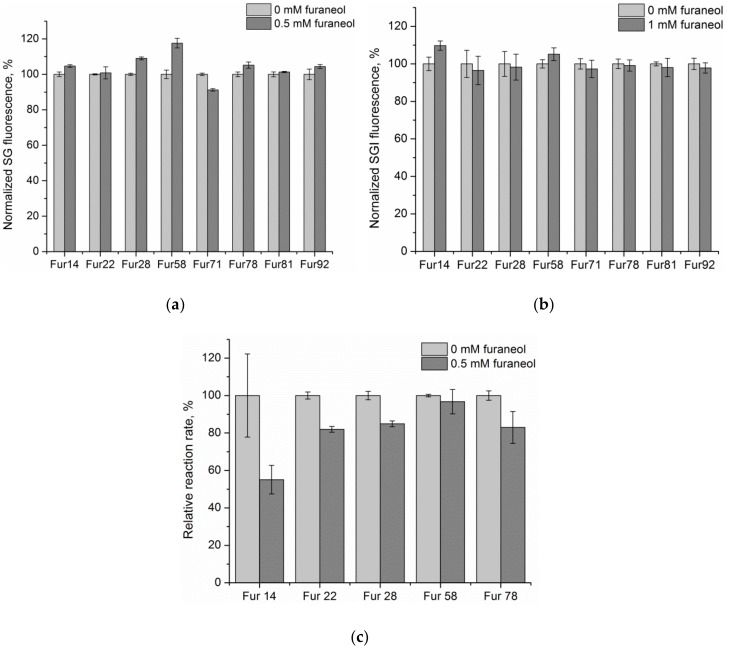
(**a**) Comparison of the ssDNA amount eluted by the buffer (0 mM furaneol, background elution) and by 500 µM of furaneol. Measurements were performed in duplicates. Background elution for each aptamer was set as 100%. (**b**) Comparison of the SGI fluorescence intercalated with the aptamer candidates, in the absence (0 mM) and in the presence of 1 mM furaneol. Measurements were performed in triplicates. Fluorescence in the absence of furaneol was set as 100%. (**c**) Comparison of the initial reaction rates of the ssDNA degradation by exonuclease I, for each aptamer candidate, in the absence of furaneol and in the presence of 0.5 mM furaneol. Measurements were performed in duplicates. Reaction rate for each aptamer, in the absence of furaneol was set as 100%. Error bars for each assay were calculated as standard deviation.

The selected candidate aptamers were chemically synthesized. Three different assays were used to estimate their ability to bind to furaneol. First, the selected oligonucleotides were hybridized onto magnetic beads covered with a capture probe and incubated with the target, at different concentrations, with the selection buffer (SB). The eluted DNA was stained by the SYBR Gold dye, and its fluorescence was recorded. Results for the highest furaneol concentration are presented in Figure 3a. Diagrams with all the concentration ranges, for each oligonucleotide, can be found in Supplementary (Figure S3). SYBR Green I assay [33] was also applied for the candidate aptamer screening. Results are presented in Figure 3b. One more binding assay, based on the protection of the ssDNA from degradation by Exonuclease I, which is provided by the target, was applied, to study the five candidate aptamers (Figure 3c). The initial rate of the exonuclease reaction was monitored by the loss of SYBR Green I dye fluorescence, during DNA degradation, in the absence or in the presence of the target.

With *p* = 0.05, the difference between the bars was statistically significant for Fur28, Fur71, Fur58, and Fur14 in Figure 3a, for Fur14 in Figure 3b, and Fur22 and Fur28 in Figure 3c. Fur_14 oligonucleotide showed doubtful results in the exonuclease I protection assay (Figure 3c). It demonstrated the maximum difference between the reaction rate, in the presence and absence of the target, but according to the t-test, this difference did not prove to be statistically significant, due to large error bars of the experimental points. Fur14 was additionally tested in exonuclease I protection assay with 200 µM furaneol, and the reaction rate in the presence of the target decreased, significantly, in comparison to the probes without furaneol (see Figure S4). Fur14 showed the best binding results in the SYBR Green I assay and the exonuclease reaction assay. In the specific elution assay, Fur_58 had the maximum elution at the highest furaneol concentration (500 µM, Figure 3a). Fur_14 exhibited moderate binding in the specific elution assay (Figure 3a). Based on a combination of the results of three assays, Fur_14 was selected as the aptamer for further characterization.

### 2.3. Aptamer Characterization

#### 2.3.1. Dissociation Constant Measurement

**Figure 4:**
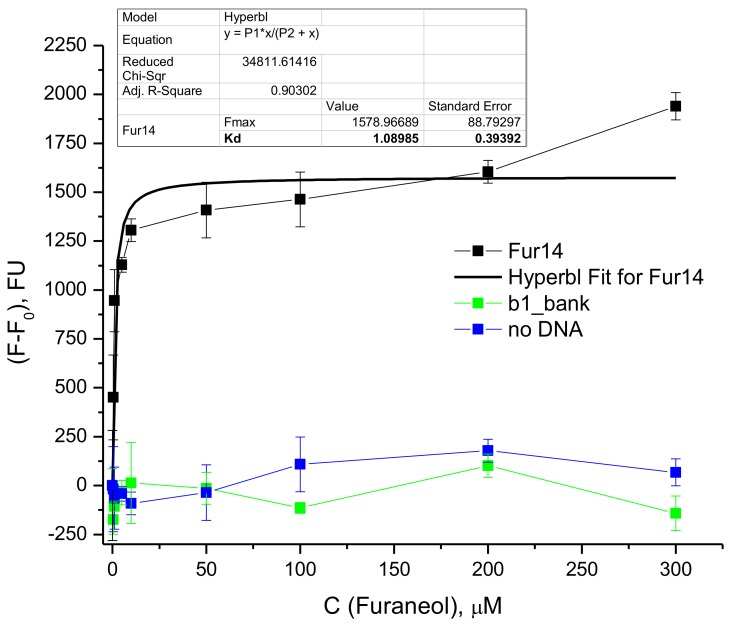
Calculation of the dissociation constant for Fur_14. Measurements were performed in triplicates. Error bars were calculated as standard deviation.

The apparent equilibrium constant for the Fur_14 oligonucleotide was determined using the SYBR Green I assay [33]. The aptamer was incubated with furaneol, at different concentrations, and the DNA was stained with the SYBR Green I fluorescent intercalating dye. Emissions of the SYBR Green I increased with the furaneol concentration. The equilibrium dissociation constant (K_D_) of the aptamer-furaneol interaction was calculated using non-linear regression (Figure 4). K_D_ was estimated to be (1.1 ± 0.4) µM.

#### 2.3.2. Aptamer Selectivity Test

**Figure 5:**
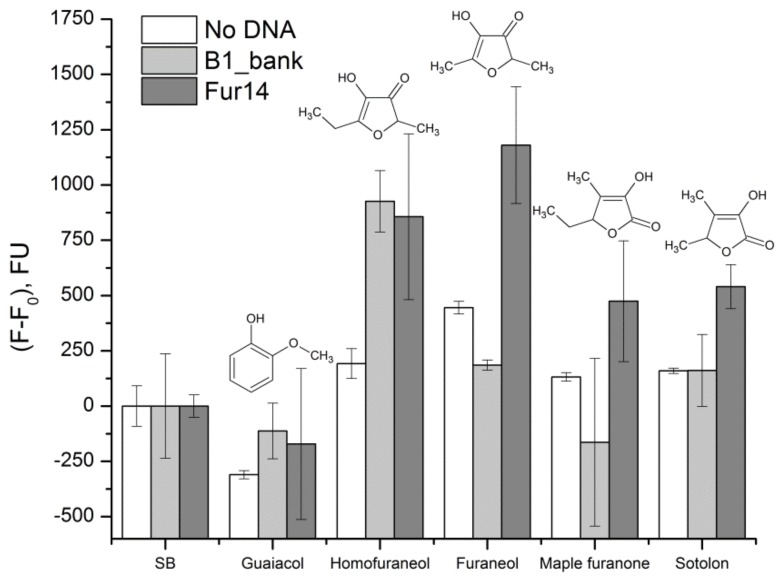
Change of the SYBR Green I fluorescence, after the incubation of Fur_14 oligonucleotide with different targets, 500 µM each. The B1_bank was used as the non-binding DNA control. Measurements were performed in triplicates. Error bars were calculated as standard deviation.

Selectivity of Fur_14 was checked by the SYBR Green I assay to compounds structurally similar to furaneol—maple furanone (5-Ethyl-3-hydroxy-4-methyl-2(5*H*)-furanone), sotolon (4,5-Dimethyl-3-hydroxy-2,5-dihydrofuran-2-one), homofuraneol (5-Ethyl-4-hydroxy-2-methyl-3(2*H*)-furanone), and guaiacol (2-Methoxyphenol). The B1_bank was used in the same assay as the non-binding DNA control. Results of the experiment are presented in Figure 5. All the targets caused change in the background fluorescence of the SYBR Green I dye. Experimental points for both Fur14 and B1_bank were corrected to this background fluorescence. Fur14 showed good selectivity to furaneol, in comparison to other compounds, some specific signal was also caused by the maple furanone and the sotolon.

### 2.4. Biosensor Development

#### 2.4.1. ISFET Fabrication and Characterization

The biosensor was fabricated using an ISFET with tantalum oxide gate dielectric. The structure was obtained earlier, where it has been fully described [34]. The subthreshold slope for the developed structures was about s = 70 mV/dec. This value was close to the theoretical limit s_0_ = 60 mv/dec.

#### 2.4.2. Aptamer Immobilization

The selected aptamer was immobilized on the Ta_2_O_5_ surface of the ISFET, using Cu-catalyzed click-chemistry [26]. For this, the 5′-end of the aptamer was modified with an alkyne label, Ta_2_O_5_ surface was covered, first, with 3-aminopropyltriethoxysilane and then with azidobutiric-N-hydroxy-succinimide, thus, producing an azide film on the surface. The alkyne-modified aptamer was “clicked” onto the transistor-sensitive surface, by the azide-alkyne cycloaddition reaction, catalyzed by the copper-containing reagent (Cu(II)-TBTA). After immobilization, the aptamer was hybridized with a short complementary oligonucleotide (5′-Biotine-GTCSpacer18-CATCGAGACTCC-3′).

#### 2.4.3. Biosensor Response

**Figure 6:**
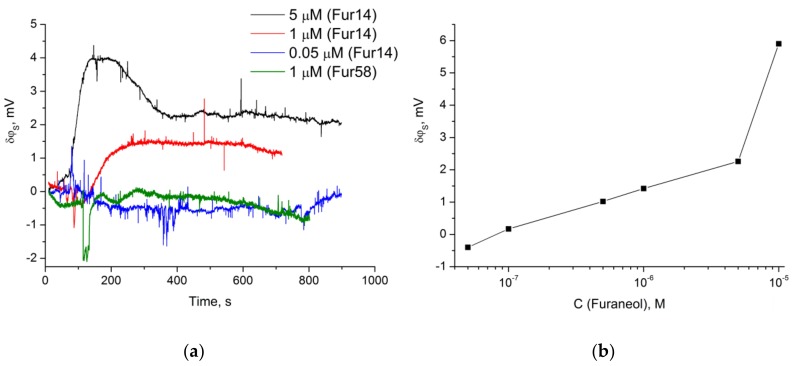
(**a**) Real-time signal of the ISFET modified with Fur14 and Fur58 oligonucleotides, to the addition of furaneol at different concentrations. (**b**) Dependence of the biosensor signal at ~450 s, after furaneol addition onto the target concentration. ISFET was modified with a Fur14 oligonucleotide.

The ISFET modified with the Fur_14 oligonucleotide, hybridized with the complementary probe, was tested for furaneol detection in a concentration range of 0.01 to 10 µM. The drain current of the transistor changed after the target addition (Figure 6a), and the response increased with the target concentration (Figure 6b). The biosensor response was detected, starting from a furaneol concentration of 0.1 µM. No response was detected on an addition of 1 µM furaneol to the ISFET modified with a Fur_58 oligonucleotide (Figure 6a).

## 3. Discussion

Selection of aptamers binding to small molecule targets is always challenging because the target immobilization creates steric hindrance and can result in a loss of binding sites. Additionally, the affinity of aptamers to small molecule targets is known to be inferior to large targets [35,36]. As mentioned above, the Capture-SELEX protocol is suitable for the selection of aptamers binding to small molecule targets, because it allows using a free target solution without the loss of possible binding sites and unwanted steric restrictions [23]. This technique was applied for isolation of an aptamer to furaneol, a volatile, small, organic substance, with an intense sweet aroma.

The B1_bank was attached to the magnetic beads covered with a special hybridization probe, complementary to the capture sequence of the library, and incubated with a free furaneol solution. During incubation with the target, oligonucleotides capable of binding with furaneol switched from the capture probe to the complex with the target, thus, passing into the solution. The eluted DNA pool was PCR-amplified, with modified primers. The elongated reverse primer served to allow subsequent ssDNA regeneration, using strands separation, by size, on the denaturing PAGE. The Cy3-labeled forward primer, enabled the DNA quantification by Cy3 fluorescence, for each eluted ssDNA pool. The enrichment of the library with sequences affine to the target, was considered to be terminated, as the amount of eluted DNA and the affinity of the eluted pool to the furaneol stopped growing. A total of thirteen cycles of selection were performed (Figure 1). The resulting DNA pool was cloned, and ninety-six plasmids were sequenced. Among them, seventy-four plasmids had inserts (see Figure S1). These insert sequences were analyzed for similarity, with a constructed phylogenetic tree (see Figure S2). Eight sequences were selected as aptamer candidates. Alignment of these sequences is presented in Figure 2. The sequences of the oligonucleotides contained conserved primer binding sites. The capture fragment of the B1_bank library also stayed intact with a small exception for Fur_14 oligonucleotide, which was missing thymine, at position 31. A CCA motif at positions 19–22 was common, for most of the sequences.

Eight aptamer candidates were chemically synthesized and screened for the ability to bind to furaneol, using three methods. The first assay was designed similar to the aptamer selection scheme and was based on the dissociation of the capture probe hybridized aptamer, in the presence of different target concentrations. The eluted DNA was quantified by the SYBR Gold staining (Figure 3a and Figure S3). In this assay Fur_58 showed the best results at the highest furaneol concentrations (500 µM), with an 18% higher fluorescent signal, compared to the background elution. Fur_14 and Fur_28 oligonucleotides also exhibited binding to furaneol, in this assay (Figure 3a).

SYBR Green I assay [33] is a simple assay, recommended for the screening of small molecule aptamers [37]. The SYBR Green I is a DNA intercalating fluorescent dye, which preferably stains the double-stranded DNA. Aptamer can change its 3D structure upon binding to the target, and this alteration can be detected by the change of the SGI fluorescence intensity. Results presented in Figure 3b indicate that the SYBR Green I fluorescence increased in the presence of the target for Fur_14.

The exonuclease protection assay is based on possible obstruction of the Exonuclease I action, by the formation of the target-aptamer complex. Exonuclease I degrades ssDNA in the 3′ to 5′ direction, with a distributive mechanism. The exonuclease reaction rate was slowed down in the presence of furaneol, for Fur22, Fur28 (Figure 2c), and Fur_14 (Figure S4).

Based on the screening of the binding ability of the aptamer candidates, Fur_14 was selected as the most promising. This oligonucleotide showed moderate results in the elution assay, but this could be explained by a poor hybridization of Fur_14, on the capture probe, due to the damage of the capture-sequence of Fur_14. The selected sequence was further characterized.

The apparent equilibrium dissociation constant for the Fur_14, measured by the SYBR Green I assay, was calculated to be (1.1 ± 0.4) µM (Figure 5). This value is good enough for small molecule aptamers [35,36]. A cross-selectivity study showed that the Fur_14 exhibits a good selectivity to furaneol, in comparison to other targets. Sotolon, an isomer of furaneol, and maple furanone also provided some specific increase of the fluorescent signal. Possibly, the ordered arrangement of methyl, hydroxyl, and oxygen substituents, in the furanone ring, contributes to the interaction of the aptamer and the target.

The selected aptamer was tested for a biosensing application. ISFET with the Ta_2_O_5_ gate served as the transducer of the biosensor. Ta_2_O_5_ was chosen as a sensing surface because of its high sensitivity and chemical stability in water solutions [38]. Fur_14 was immobilized onto the ISFET-sensitive surface, using copper-catalyzed click-chemistry. For this, the aptamer was labeled with alkyne at the 5′ end, and the Ta_2_O_5_ surface was functionalized with an azide layer, using self-assembled monolayers technique.

All the aptamers isolated with the Capture-SELEX protocol are intended to demonstrate the structure-switch between the capture-probe and the target, and other non-switching aptamers are expected to be discarded, during the partition step of each selection cycle. ISFET is capable of detecting DNA-DNA hybridization-dehybridization [30], and namely, detecting the structure-switch of the aptamer, upon addition of the target, resulting in a dehybridization of the capture probe. Hence, Fur_14 immobilized on the ISFET was hybridized with the capture probe used during the selection. Indeed, the drain current of the transistor changed after addition of 1 µM furaneol, in comparison to the buffer addition, and no response to furaneol was detected for the Fur_58 oligonucleotide that was also tested in the same experiment. The biosensor response was proportional to the furaneol concentration. The developed aptasensor can detect as low as 0.1 µM of furaneol.

Furaneol is usually analyzed by hardware-equipped and time-consuming chromatographic methods [3,10,15,16,17]. The concentration of furaneol in the natural samples varies from low nanomolar values of 0.3–9 nM, in tomatoes [10], to micromolar 1.5–7 µM, in wines [3]. For most, the sample extraction and concentration steps are required for the analysis [3,10,15,16,17]. To our knowledge, neither aptamer to furaneol nor any biosensor for the furaneol detection has yet been reported. Here, we showed that the reported aptamer could be used for furaneol analysis, and this could find application in food science and the food industry.

## 4. Materials and Methods

### 4.1. Materials

Sodium chloride, magnesium chloride, potassium chloride, calcium chloride, 2-amino-2-(hydroxymethyl)-1,3-propanediol (Trizma base), hydrochloric acid, 2,5-dimethyl-4-hydroxy-3(2H)-furanone (furaneol), 5-Ethyl-3-hydroxy-4-methyl-2(5H)-furanone (maple furanone), 4,5-Dimethyl-3-hydroxy-2,5-dihydrofuran-2-one (sotolon), 5-Ethyl-4-hydroxy-2-methyl-3(2H)-furanone (homofuraneol), 2-Methoxyphenol (guaiacol), and ethylenediaminetetraacetic acid disodium salt dihydrate (EDTA) were purchased from Sigma-Aldrich (Munich, Germany). Taq DNA polymerase, dNTPs solution, TAE and TBE electrophoresis buffers, DNA extraction kits, DNA cloning kit, Exonuclease I, and the *Escherichia coli* DH5α strain were purchased from Thermo Fisher Scientific (Waltham, MA, USA). SYBR Gold dye was purchased from Invitrogen (Thermo Fisher Scientific). SYBR Green I dye was obtained from Lumiprobe Ltd., (Moscow Russia). Streptavidin-coated magnetic beads were supplied by New England Biolabs (Ipswich, MA, USA).

The single-stranded DNA library B1_bank was synthesized by DNA Synthesis Ltd., Moscow, Russia. All primers for DNA amplification were ordered from the DNA Synthesis Ltd., Moscow, Russia. The B1_cap was either purchased from DNA Synthesis Ltd., Moscow, Russia or from Microsynth AG (Balgach, Switzerland). Candidate aptamer sequences were synthesized by Evrogen Ltd. (Moscow, Russia).

### 4.2. Aptamer Selection

The protocol for aptamer selection was adopted from the Capture-SELEX procedure reported by Stoltenburg et al. [23] and is detailed in [28]. 1 mM furaneol in Selection Buffer (SB: 100 mM NaCl, 20 mM Tris-HCl [pH 7.6], 2 mM MgCl_2_, 5 mM KCl, 1 mM CaCl_2_) served as the target for the selection. The B1_bank ssDNA library (5′-CGACCAGCTCATTCCTCA-N10-GGAGTCTCGATG-N40-GGATCCGAGCTCACCAGTC-3′) served as the starting library for the selection.

In brief, the biotin-labeled B1_cap oligonucleotide (5′-Bio-GTC-Spacer18-CATCGAGACTCC-3′), complementary to the capture sequence of the B1_bank library, was immobilized on streptavidin coated magnetic beads. 3 nmol of the ssDNA library B1_bank was then hybridized with the B1_cap probe and, thus, fixed reversibly onto the magnetic beads. 5 mg of the streptavidin-covered magnetic beads, capable of binding to 2.5 pmol of the biotinilated ssDNA, were used for the first selection round, and 320 µg, for the subsequent rounds. The unbound ssDNA was washed away by rinsing the particles with SB, seven times. Weakly-bound sequences were eliminated by incubating the particles at 28 °C for 15 min, followed by seven washes with SB. The beads covered with the ssDNA library were incubated with the 1 mM furaneol solution, in the SB. Before each incubation with the target, magnetic nanoparticles, with hybridized ssDNA, were incubated in the same volume of SB, to monitor the background DNA elution. The elution volume was 200 µL, for the first, and 50 µL, for the other rounds. The eluted DNA was PCR-amplified with the use of Cy3-labelled forward primer B1_for_Cy3 (5′-Cy3-CGACCAGCTCATTCCTCA-3′) and extended with polyA20 tail reverse primer B1_rev_A (5′-AAAAAAAAAAAAAAAAAAAA-HEGL-GACTGGTGAGCTCGGATCC-3′). The ssDNA was amplified with five cycles, in the first round and by ten cycles, for all the subsequent rounds. The dsDNA product, with chains of different lengths, was denatured and the DNA strands were separated in 12% denaturing PAGE. The Cy3-labeled sense ssDNA strand was cut off from the gel and extracted, using the crush and soak protocol [39], followed by an ethanol/LPA DNA precipitation. The entire obtained ssDNA pool was quantified using Cy3 absorbance and used for the next round of aptamer selection. Starting from the second round, the amount of eluted ssDNA was monitored using Cy3 fluorescence, measured by the TECAN microplate reader (TECAN, Austria). Target concentration (1 mM), time (30 min), and temperature (2 °C) of elution, by buffer and by target, and the washing procedure, were the same for all selection rounds. Thirteen rounds of selection were performed.

### 4.3. Cloning and Sequencing

After the final SELEX round, ssDNA was amplified using the non-modified primers B1_for (5′-CGACCAGCTCATTCCTCA-3′) and B1_rev (5′-GACTGGTGAGCTCGGATCC-3′). The dsDNA product was purified with a PCR purification kit and cloned using a CloneJet DNA cloning kit. The *Escherichia coli* DHα strain rubidium competent cells were transformed with the ligation mixture. Plasmids were extracted with a GeneJet plasmid DNA extraction kit. The inserts were Sanger sequenced by Syntol Ltd., Russia. Sequences were processed using the BioEdit and Unipro UGENE software. A built-in algorithm of the Ugene, PHYLIP Neighbor-Joining method was used to create phylogenetic tree of the sequences.

### 4.4. Screening the Aptamer Candidates’ Binding Ability by Elution from Magnetic Beads

200 pmol of each aptamer candidate was denatured and hybridized with 200 μg of magnetic beads covered with the capture probe, as described above. After the DNA hybridization, the magnetic beads were washed properly from the unbound and weakly-bound DNA and divided into ten equal probes. The probes in the duplicate were incubated with 0, 0.5, 5, 50 and 500 µM of the furaneol solution in the SB. After 30 min of incubation with gentle shaking, the magnetic beads were separated using a magnetic rack. 35 µL of the solution was mixed with 65 µL of 3× SYBG Gold solution in SB and incubated for 20 min, at room temperature. The fluorescence of the SYBR Gold (excitation at 495 nm, emission at 537 nm) was recorded using the TECAN microplate reader (TECAN, Austria).

### 4.5. SYBR Green I Assay

Each aptamer candidate was annealed, as described above. To test the binding ability of aptamer candidates, a premix containing 10 nM of the aptamer and 1.25 mM of the extra MgCl_2_, in SB, was prepared for each oligonucleotide. The premix was pipetted by 85 µL to the microplate wells containing 10 µL, of SB, or 10 mM furaneol solution in SB, each in triplicates. The mix was incubated for 30 min, at room temperature, and then 5 µL of the 5× SYBR Green I solution in SB, was added to each well. After 2 h of incubation, the fluorescence of the SYBR Green I (excitation at 480 nm, emission at 520 nm) was recorded using the TECAN microplate reader (Tecan Group Ltd, Männedorf, Switzerland). Measurements were performed in duplicates.

The SYBR Green I assay was applied for the determination of the dissociation constant and cross-selectivity study for Fur_14 oligonucleotide. The DNA concentration of 5 nM was fixed. The B1_bank library was used as the non-binding control. Measurements were performed in triplicates. Furaneol concentration for the K_D_ measurement was varied from 0.5 to 300 µM, the fluorescent signal was plotted against the furaneol concentration. *K*_D_ was calculated by fitting the binding data to a one-site saturation equation (*Y* = (*F* − *F*_0_) × *X*/(*K*_D_ + *X*), where F is the sample fluorescence, *F*_0_ is fluorescence of the probe, without furaneol) in the OriginPro software 8.5 (OriginLab Corp., Northampton, MA, USA). Cross-selectivity of the Fur_14 oligonucleotide was studied using 500 µM solutions of homofuraneol, furaneol, maple furanone, and sotolon, in SB.

### 4.6. Exonuclease Protection Assay

Each aptamer candidate was dissolved in SB to a final concentration of 1 × 10^-6^ M and denatured at 92 °C for 8 min, then cooled on ice. A master mix containing 1 × 10^-8^ M of denatured oligonucleotide and 1.25 mM MgCl_2_, in SB, was prepared and pipetted by 89 µL to the wells of white 96 well microplate (Greiner, Germany). 10 µL of the furaneol solution, in SB, was added to a final concentration of 0.5 mM, 10 µL of SB were added for the negative control measurements, measurements were performed in duplicates. The plate was incubated for 30 min at the Eppendorf Thermomixer Comfort (Germany), with a gentle rotation. Then 5 µL of the 1× SYBR Green I dye solution, in SB, was added to each well, and the plate was incubated for five more minutes. Five units of the Exonuclease I was added to each well, and the fluorescent signal (excitation at 480 nm, emission at 520 nm) was recorded for 1 h, using the TECAN microplate reader (Tecan Group Ltd, Männedorf, Switzerland). Slope ratio for each reaction was determined from the kinetic curves, using linear regression, in the OriginPro software 8.5 (OriginLab Corp.).

### 4.7. ISFET Fabrication

n-type ISFET was fabricated using standard 1.2-μm FD SOI CMOS technology on 4′-SOI SIMOX wafers (p-type), with a 0.18-µm-thick active layer, 0.38-µm-thick insulating layer, and 12–22 Ohm.cm resistivity in the SMC “Technological Centre”, Russia. Active layer was further thinned to 100 nm by oxidation. After the formation of polysilicon gate, 50 nm of tantalum was deposited by the PVD onto 10 nm SiO_2_ gate dielectric. Then, the Ta_2_O_5_ was obtained by thermal oxidation of the tantalum film at 850 °C, in oxygen, for 10 min, and a standard metallization step was carried out. In this paper the transistor with a channel width of 6 μm and length of 100 μm was used. Pt wire was used as a reference electrode.

### 4.8. Aptamer Immobilization

The selected aptamer was immobilized on the Ta_2_O_5_ surface of the transistor, using Cu-catalyzed click-chemistry [26]. For this, the 5′-end of the aptamer was modified with alkyne label, Ta_2_O_5_ surface was covered, first, with 3-aminopropyltriethoxysilane (40 min in 3% silane in methanol) and then with azidobutiric-Nhydroxy-succinimide, thus, producing an azide (N3) film on the surface (4 h in 4 mM solution in 0.1 M Tris/HCl buffer, pH 8.6). The alkyne-modified aptamer was “clicked” onto the transistor-sensitive surface, by the azide-alkyne cycloaddition reaction, catalyzed by the copper-containing reagent (Cu(II)-TBTA): aptamer 1 μM, Cu(II) 50 μM, ascorbic acid 2 mM, incubation overnight. After immobilization, the aptamer was hybridized with the 20 pmol/μL short complementary oligonucleotide 5′-Biotine-GTC-Spacer18-CATCGAGACTCC-3′, over 2 h.

### 4.9. ISFET Measurements

All measurements of the ISFETs were carried out in a well-like structure which was made of wax, with 3D-printing technology [40].

The ISFET was operated in a subthreshold mode; for this, the *I*-*V*_G_ curves (*V*_DS_ = 0.1 *V*) were recorded. The threshold voltage *V*_T_ and subthreshold swing S were used. Time-dependent changes in the current *I*_DS_ (*V*_G_ = const) were recorded and the surface potential change was used as a signal [28].

Measurements began with the determination of the operating mode—the ISFET with well-like structure contained 35 μL of the selection buffer. Then time-dependent changes in the I_DS_ (V_G_ = const) were recorded and during the recording, 1.5 μL of the selection buffer or furaneol, at different concentrations, was added to initiate the dehybridization of the probe from the immobilized aptamer. The fact of dehybridization was detected by the transistor.

For data processing, surface potential calculations (*I*_DS_ → ∆*φ*) were made. As a signal from each measurement, a value of difference between the control (time-dependent changes with addition of 1.5 μL of SB) and sample, after ~450 s of furaneol addition, was used for the calculations.

## Figures and Tables

**Table 1:** The ssDNA load and elution during the aptamer selection.

SELEX Cycle Number	ssDNA Input, pmol ^1^	Amount of ssDNA Eluted by Buffer, pmol ^2^	Amount of ssDNA Eluted by 1 mM Furaneol, pmol ^2^
1	not available	not detected	not detected
2	3.2	0.11	0.10
3	not detectable	0.03	0.04
4	0.9	0.17	0.16
5	7.7	0.31	0.29
6	1.5	0.39	0.36
7	11.5	0.35	0.40
8	1.8	0.69	0.79
9	4.0	0.31	0.41
10	12.0	0.62	1.40
11	2.1	0.03	0.04
12	1.3	0.003	0.005
13	2.5	0.05	0.09

^1^ Quantified by Cy3 label absorbance; ^2^ Quantified by Cy3 label fluorescence.

## Supplementary Materials

The following are available online.
