# Supplementary material for: Selection, Characterization, and Application of ssDNA Aptamer against Furaneol
Source: Molecules. 2018 Nov 30;23(12):3159. doi: 10.3390/molecules23123159 (PMC6320952; doi:10.3390/molecules23123159)
Supplement: Supplementary file 1 [file molecules-23-03159-s001.pdf]

Electronic Supporting Material on the Molecules publication entitled

Selection and characterization of ssDNA aptamer against furaneol

Natalia Komarova [1,*], Mariia Andrianova [1], Sergey Glukhov [1], and Alexander Kuznetsov [1]

[1] Scientific-Manufacturing Complex Technological Centre, 1–7 Shokin Square, Zelenograd, Moscow 124498, Russia; kae@tcen.ru

* Correspondence: nat.v.kom@gmail.com; Tel.: +7-926-302-7969

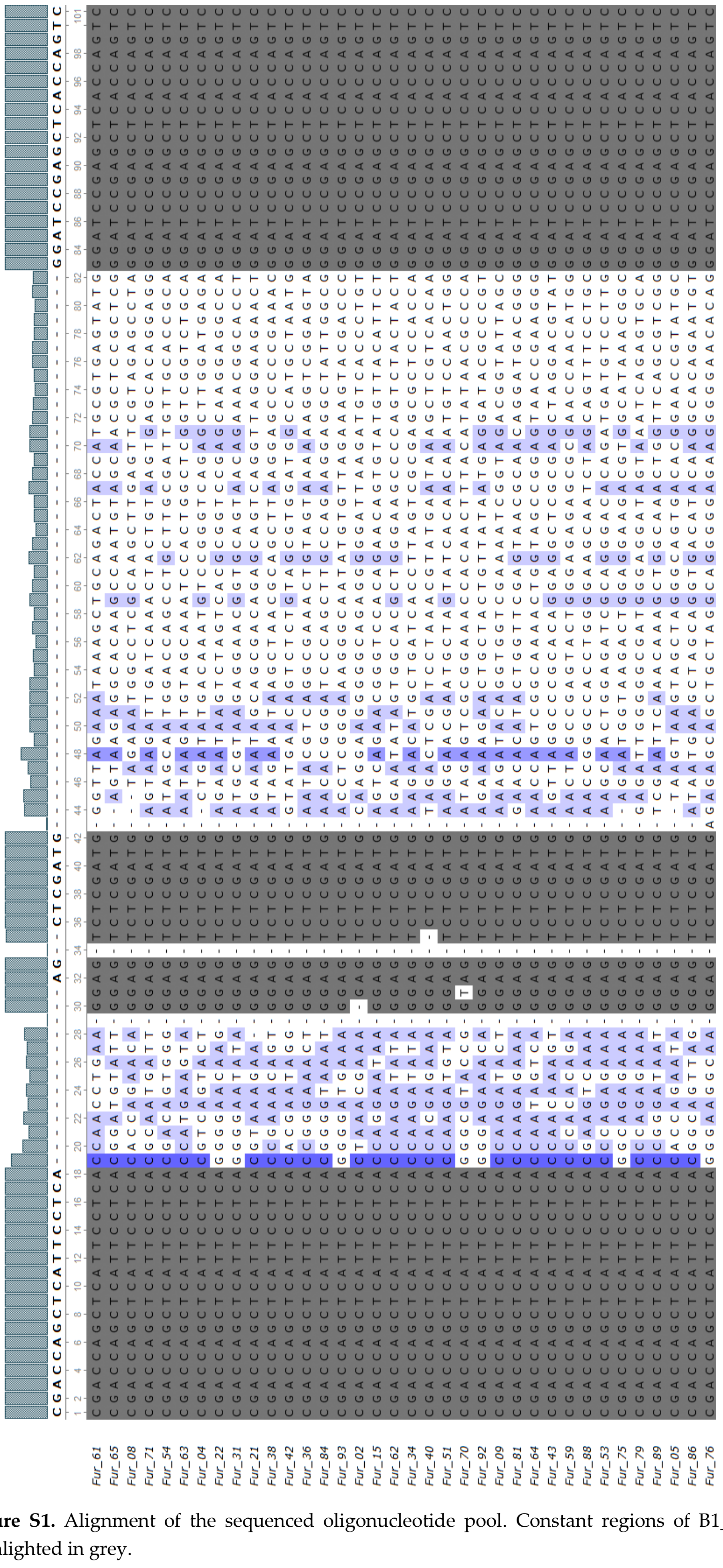

Figure S1. Alignment of the sequenced oligonucleotide pool. Constant regions of B1_bank library are highlighted in grey.

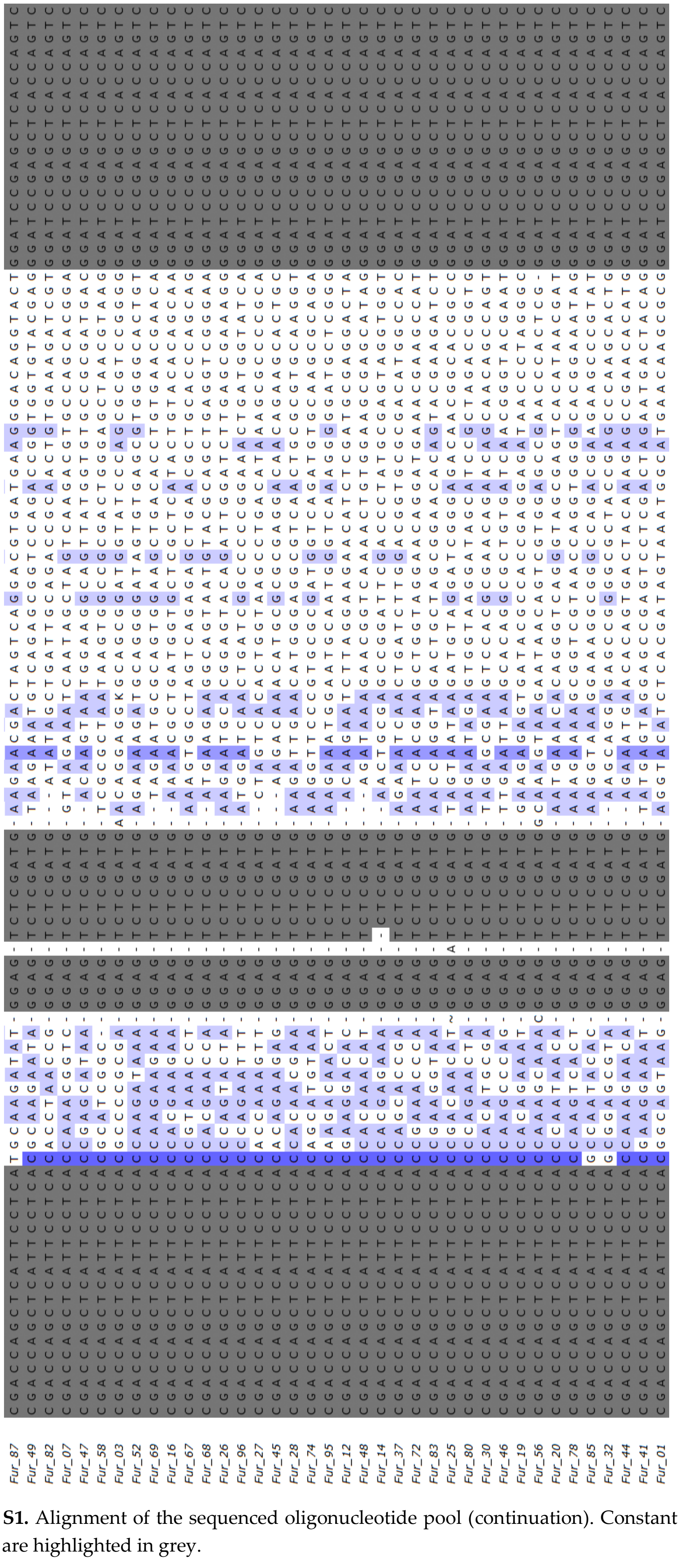

Figure S1. Alignment of the sequenced oligonucleotide pool (continuation). Constant regions of B1_bank library are highlighted in grey.

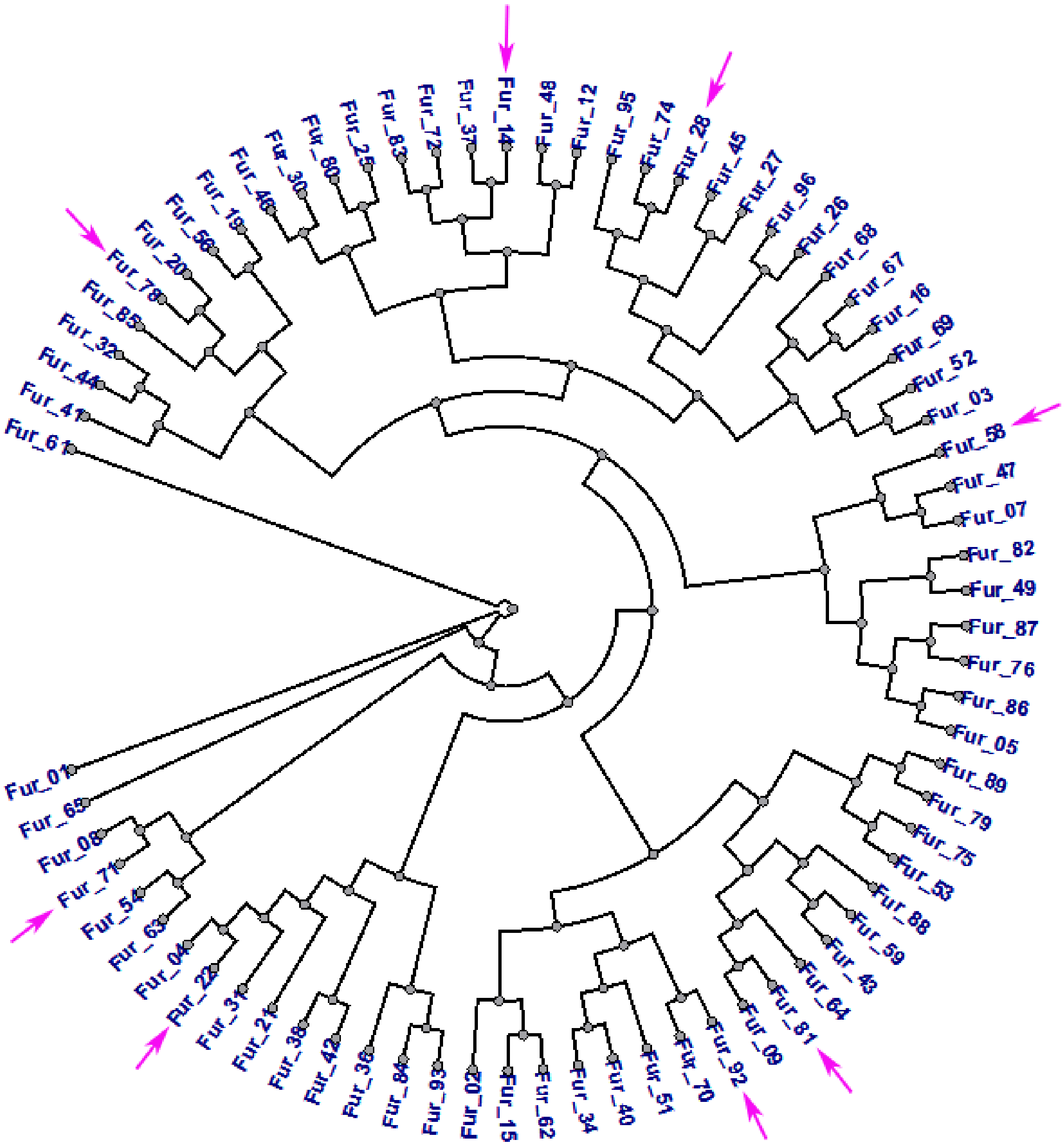

Figure S2. Phylogenetic tree of the sequenced pool of oligonucleotides.

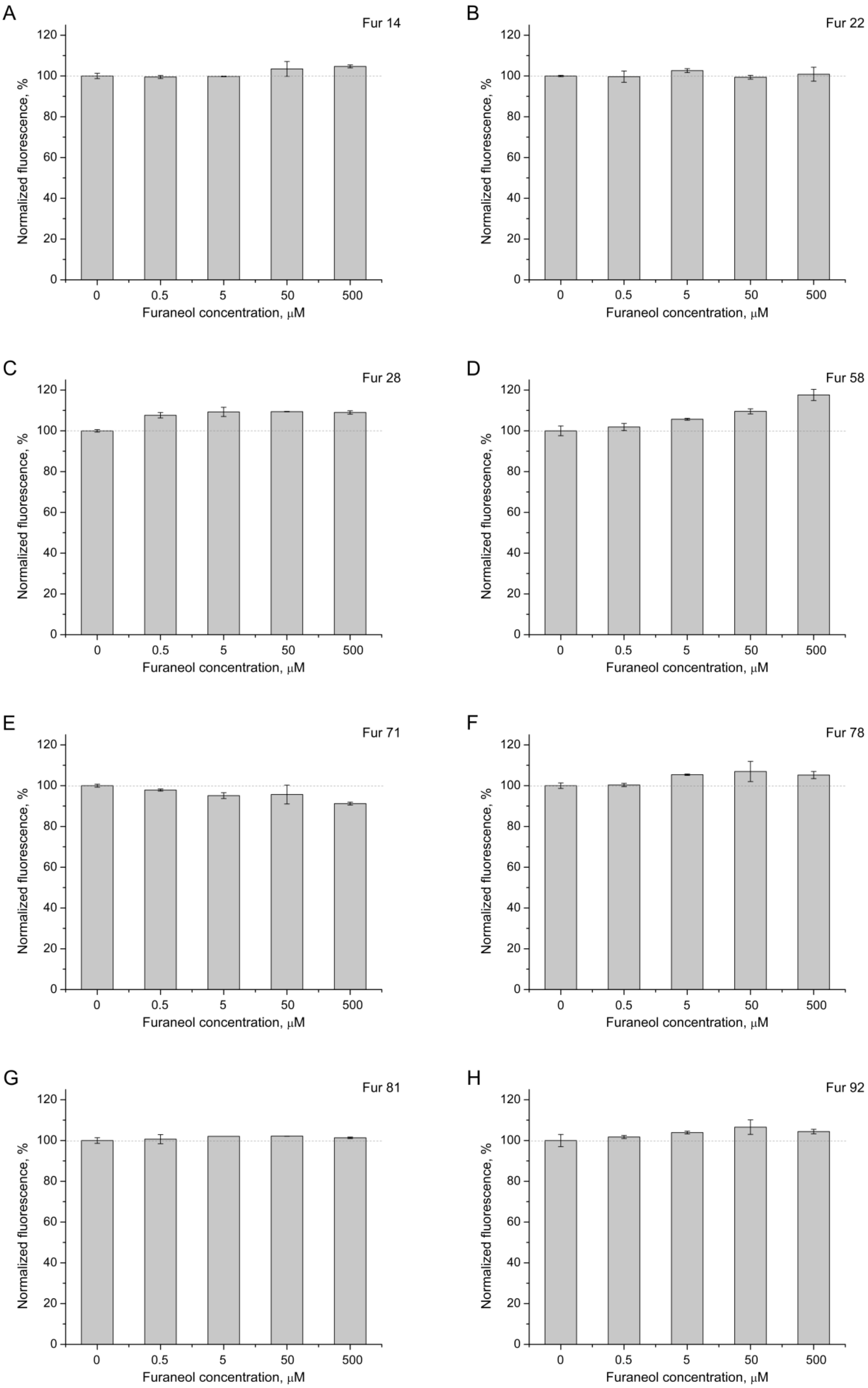

Figure S3. Results of elution-based binding assay for candidate aptamers.

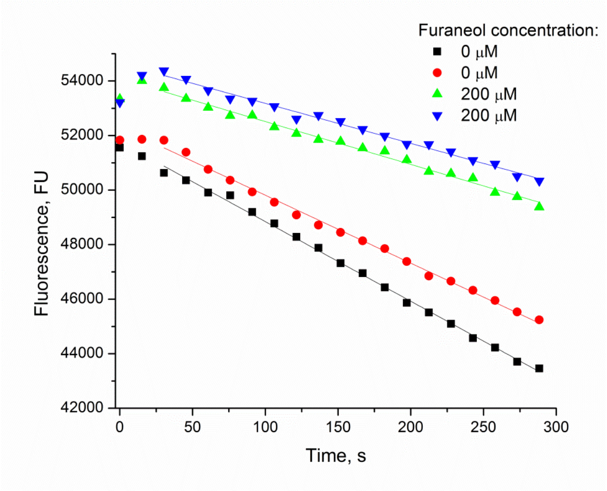

(**a**)

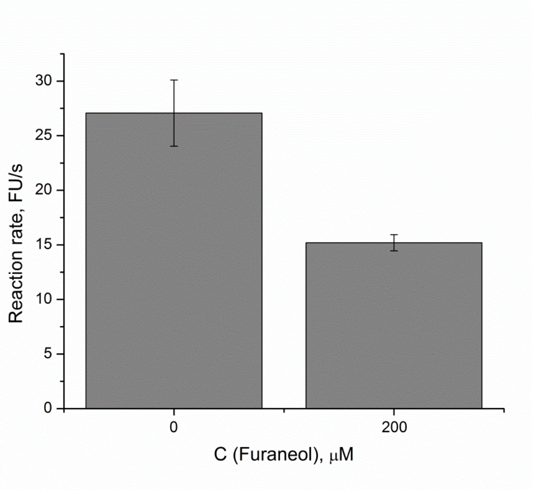

(**b**)

Figure S4. (**a**) Time dependence of fluorescent signal during digestion of Fur14 oligonucleotide by Exo I in the absence of furaneol and in the presence of 200 μM furaneol; (**b**) Comparison of exonuclease reaction rate for Fur14 the absence of furaneol and in the presence of 200 μM furaneol. Measurements were performed in duplicate, error bars were calculated as standard deviation. The difference between the bars proved to be statistically significant with p=0.05.
